# *Staphylococcus aureus* Panton-Valentine Leukocidin Is a Very Potent Cytotoxic Factor for Human Neutrophils

**DOI:** 10.1371/journal.ppat.1000715

**Published:** 2010-01-08

**Authors:** Bettina Löffler, Muzaffar Hussain, Matthias Grundmeier, Michaela Brück, Dirk Holzinger, Georg Varga, Johannes Roth, Barbara C. Kahl, Richard A. Proctor, Georg Peters

**Affiliations:** 1 Institute of Medical Microbiology, University Hospital of Münster, Germany; 2 Interdisciplinary Center of Clinical Research, University Hospital of Münster, Germany; 3 Institute of Immunology, University Hospital of Münster, Germany; Dartmouth Medical School, United States of America

## Abstract

The role of the pore-forming *Staphylococcus aureus* toxin Panton-Valentine leukocidin (PVL) in severe necrotizing diseases is debated due to conflicting data from epidemiological studies of community-associated methicillin-resistant *S. aureus* (CA-MRSA) infections and various murine disease-models. In this study, we used neutrophils isolated from different species to evaluate the cytotoxic effect of PVL in comparison to other staphylococcal cytolytic components. Furthermore, to study the impact of PVL we expressed it heterologously in a non-virulent staphylococcal species and examined *pvl*-positive and *pvl*-negative clinical isolates as well as the strain USA300 and its *pvl*-negative mutant. We demonstrate that PVL induces rapid activation and cell death in human and rabbit neutrophils, but not in murine or simian cells. By contrast, the phenol-soluble modulins (PSMs), a newly identified group of cytolytic staphylococcal components, lack species-specificity. In general, after phagocytosis of bacteria different *pvl*-positive and *pvl*-negative staphylococcal strains, expressing a variety of other virulence factors (such as surface proteins), induced cell death in neutrophils, which is most likely associated with the physiological clearing function of these cells. However, the release of PVL by staphylococcal strains caused rapid and premature cell death, which is different from the physiological (and programmed) cell death of neutrophils following phagocytosis and degradation of virulent bacteria. Taken together, our results question the value of infection-models in mice and non-human primates to elucidate the impact of PVL. Our data clearly demonstrate that PVL acts differentially on neutrophils of various species and suggests that PVL has an important cytotoxic role in human neutrophils, which has major implications for the pathogenesis of CA-MRSA infections.

## Author Summary

*Staphylococcus aureus* can cause serious diseases, including necrotizing pneumonia, which often affects young immunocompetent patients and has a high lethality rate. Several clinical studies demonstrated a clear association between this form of pneumonia and *S. aureus* strains carrying the gene for the pore-forming toxin Panton-Valentine leukocidin (PVL). However, laboratory work, which mainly used murine disease models, has created very contrasting results and often fails to show a pathogenic role for PVL. In this study, we demonstrate that the expression of PVL by staphylococcal strains confers strong and rapid cytotoxic activity against neutrophils. However, this action was basically restricted to human cells and could not be reproduced in murine or Java monkeys’ cells. These results indicate that infection-models in mice and in non-human primates fail to replicate the pathogenic activity of PVL seen in human cells. Our data with human neutrophils clearly show that PVL has a major cytotoxic effect, as the release of PVL by staphylococcal strains caused rapid and premature cell death, which is different from the physiological (and programmed) cell death of neutrophils following phagocytosis and degradation of virulent bacteria. These results have important implications especially for infections with CA-MRSA strains, which often carry the gene for PVL and have spread widely in the community.

## Introduction

*Staphylococcus aureus* is an important human pathogen that can cause serious diseases [1]. In the last few years, there was a dramatic increase in the incidence of community-associated methicillin-resistant *S. aureus* (CA-MRSA) infections in otherwise healthy individuals and resistance to multiple antibiotic classes largely limits therapeutic options. Especially the MRSA strain USA300 has widely spread within the United States and has become the cause of more unusually severe diseases, including necrotizing pneumonia, skin infections, osteomyelitis and necrotizing fasciitis [2],[3]. Necrotizing pneumonia seems to be a specific disease entity and often follows infection with influenza virus [4],[5]. To combat these life-threatening infections, there is a need to better understand the bacteria-host interaction and virulence factors involved.

Clinical studies propose the exotoxin Panton-Valentine leukocidin (PVL) as a crucial virulence factor in necrotizing diseases [4],[6]. PVL is a two component pore-forming toxin, which mainly acts on neutrophils [7]. It is expressed by only a small percentage of *S. aureus* wild-type isolates (2–3%) [8], but it is highly prevalent in *S. aureus* strains isolated from necrotizing infections [4],[6]. However, several studies that used a diversity of animal models have created conflicting results concerning the role of PVL. One study, applying a mouse acute pneumonia model, suggests PVL as major virulence factor [9]. By contrast, other groups fail to detect a pathogenic function of PVL in murine lung and skin infections and in cell culture experiments, but demonstrate a predominant role of α-hemolysin (α-toxin) and a possible relevance of the bacterial surface protein A (Spa) [10]–[12]. Both factors are expressed at high prevalence among clinical isolates and are considered to contribute to various disease entities [1],[13],[14]. Yet, when a rabbit bacteremia model was used, a transient effect of PVL in the acute phase of infection could be demonstrated [15]. Furthermore, a recent study identified a group of *S. aureus* peptides, the phenol-soluble modulins (PSMs), with strong cytolytic activity on human neutrophils. As PSMs are released at high concentrations by CA-MRSA strains and contribute to disease development in murine models, the authors propose that PSMs account for the enhanced virulence of CA-MRSA [16].

However, there is some evidence that the actions of *S. aureus* toxins can be strongly dependent on the animal species used, which should be analysed in detail to better interpret disease-models. In particular, the host cell response to PVL may be species-specific [17], whereas the effects of other staphylococcal factors, such as PSMs, might be species-independent. In this study, we used polymorphonuclear cells (neutrophils) from different species including humans, mice, rabbits and monkeys to test the effect of several virulence factors. As neutrophils are the major defending cells against bacterial invasion, their excessive cell death most likely largely promotes disease development.

## Results

### The effect of purified *S. aureus* virulence factors on neutrophils from different species

**Figure 1:**
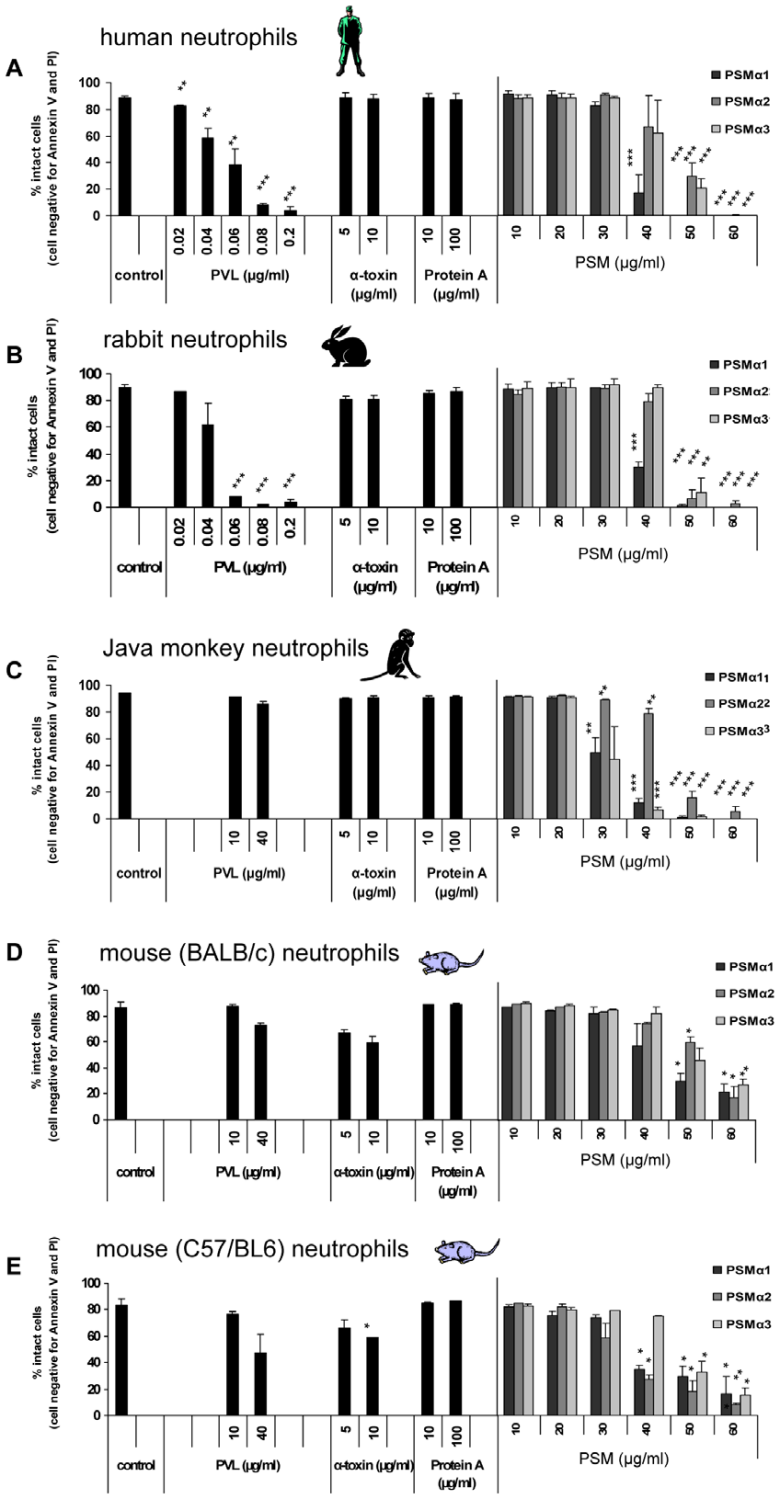
The cytolytic effect of purified *S.aureus* virulence factors on neutrophils from different species. Neutrophils from different species, including human (**A**), rabbit (**B**), Java monkey (**C**), BALB/c mice (**D**), C57/BL6 mice (**E**) were freshly isolated and 1×10^6^ 0.5 ml^−1^ cells were incubated with increasing doses of PVL, α-toxin, protein A or PSMs (PSMα1, PSMα2, PSMα3), respectively. PVL: 0.02–0.2 µg/ml (0.5–5 nM); α-toxin: 5, 10 µg/ml (150, 300 nM); protein A: 10, 100 µg/ml (0.238, 2.38 µM); PSMs: 10–60 µg/ml (4–24 µM). Neutrophils were stimulated for 1 h and then cells were washed, stained with annexin V and propidium iodide (taking another hour) and then cell death was measured by flow cytometry. The values represent the mean ± SEM of at least three independent experiments. * P≤0.05, ** P≤0.01, *** P≤0.001 (independent t-test comparing the rate of intact cells between control and stimulated cells). Taking of blood samples from humans and animals were approved by the local ethics committee.

First, we challenged human neutrophils with purified *S. aureus* components, including PVL, α-toxin, protein A and PSMs. For PVL, doses≥40 ng/ml (0.04 µg/ml) were sufficient to induce cell damage (Figure 1A). Cell death occurred rapidly, within 1 h, and most likely due to necrosis, as we could not detect characteristic apoptotic features (Figure S2) [18]. In contrast to PVL, α-toxin or protein A did not cause cell death, even when applied at high concentrations, which have pro-inflammatory or cytotoxic effects in other cell types [19],[20]. As recently published [16], three different forms of *S. aureus* PSMs (PSMα1, PSMα2, PSMα3) were able to provoke cell-lysis. However, cell death induction required relatively high doses of PSMs (≥40 µg/ml) in comparison to PVL (≥40 ng/ml) (Figure 1A). In previous studies, the impact of PVL was mainly tested on human or rabbit neutrophils, as cells from both species were reported to be susceptible to PVL [17]. In line with published data, we found similar responses of human and rabbit neutrophils to low doses of PVL (Figure 1B). The action of PVL appears to be tightly restricted to these species, as neutrophils isolated from Java monkeys (*Macaca fascicularis*, *cynomolgus*), the most commonly used non-human primate in biomedical research, were not killed in response to PVL (Figure 1C). In recent reports, models of severe staphylococcal infections were mainly performed in the murine strains BALB/c or C57/BL6 [9],[10]. However, murine neutrophils from both strains were largely resistant to PVL (Figure 1D, E), irrespective of their maturation and inflammatory state (Figure S3).

**Figure 2:**
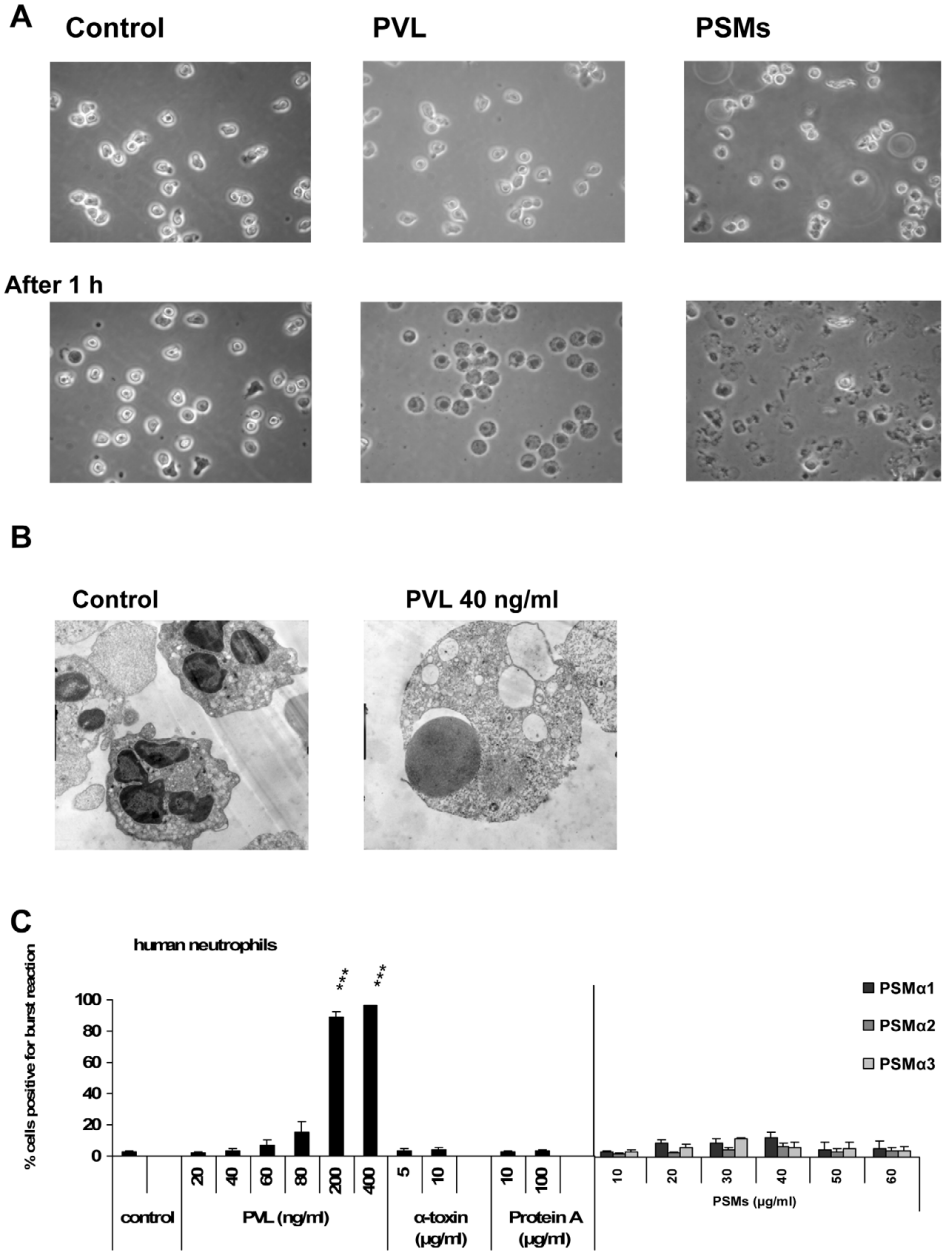
Differences between PVL- and PSMs-induced cell death. Human neutrophils were freshly isolated and stimulated with staphylococcal components as described in figure 1. Neutrophils were stimulated for 1 h with PVL (80 ng/ml) or PSMs (60 µg/ml) and cells were analyzed by light microscopy with a live cell imaging system (**A**). Neutrophils were stimulated for 1 h with PVL (40 ng/ml) and processed for electron microscopy (**B**). Cells were stimulated for 10 min and an oxidative burst reaction was determined by a burst-test (Orpegen Pharma). The values represent the mean ± SEM of at least three independent experiments. * P≤0.05, ** P≤0.01, *** P≤0.001 (independent t-test comparing the rate of burst reaction between control and stimulated cells; **C**).

In contrast to PVL, all PSM-types tested (PSMα1–3) lysed neutrophils from different species at concentrations≥40 µg/ml, indicating that the actions of PSMs apparently lack species-specificity (Figure 1A–E). Further on, we detected additional differences between PVL- and PSM-induced cell death. Incubation with PVL caused changes in cell morphology, including rounding and swelling of cells and nuclei (Figure 2A, 2B), which persisted for several hours (data not shown). By contrast, PSM-stimulated cells were rapidly destroyed without characteristic changes in morphology (Figure 2A). In PVL-treated neutrophils, an oxidative burst reaction (Figure 2C) and pro-inflammatory activation (Figure S4) accompanied cell death induction, whereas incubation with PSMs did not cause an oxidative burst (Figure 2C). These results point to completely different mechanisms of action provoked by the *S. aureus* cytotoxic components PVL and PSMs.

### The effect of live bacteria, which differ in virulence factor expression, on human neutrophils

**Figure 3:**
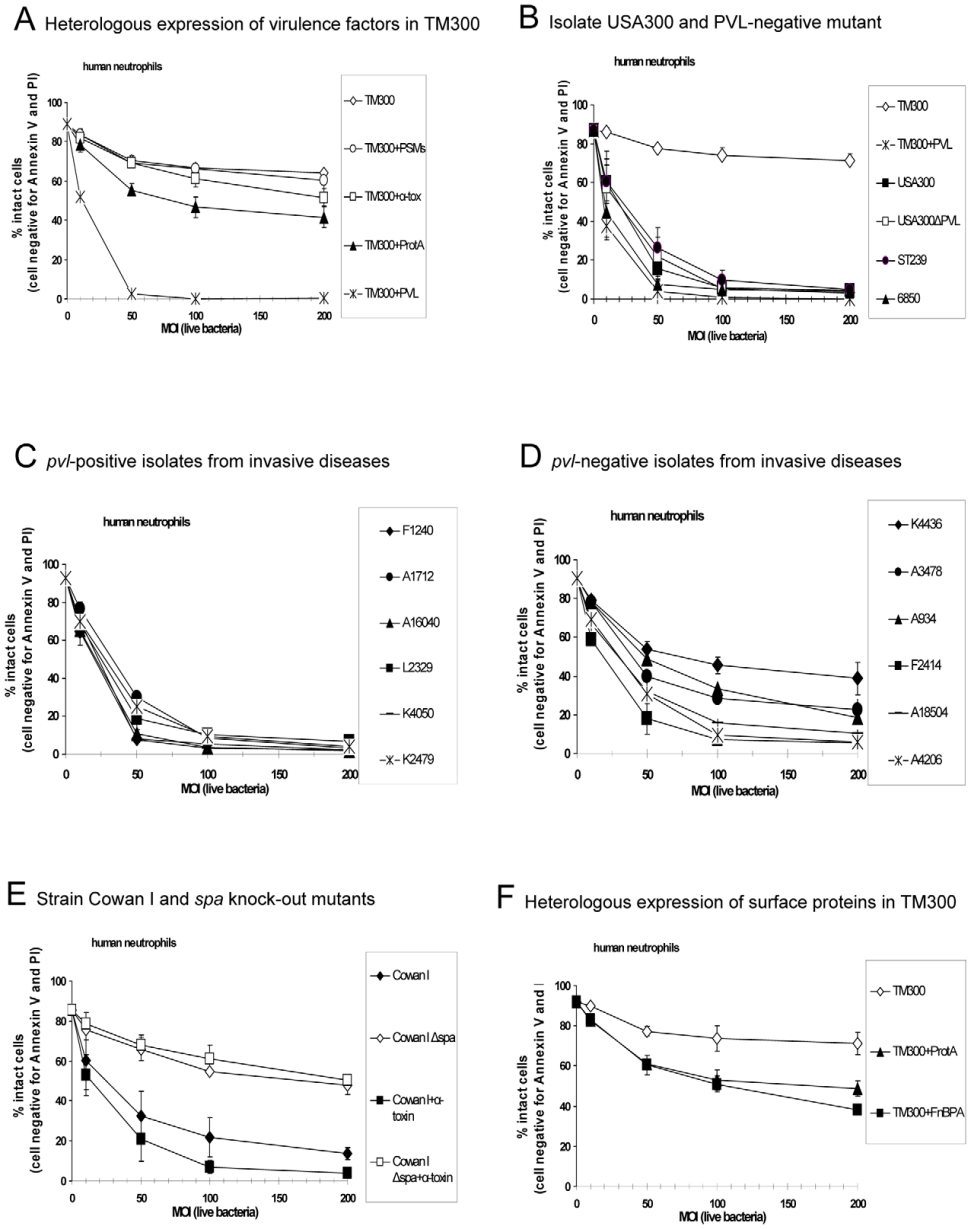
The impact of PVL expression on human neutrophil survival. Human neutrophils were freshly isolated and 1×10^6^ 0.5 ml^−1^ cells were incubated with live bacteria, which were grown in overnight cultures and used for stimulating cells at an multiplicity of infection (MOI 10–200) as indicated. In these experiments we used heterologous expression strains of TM300 and Cowan I (**A, E, F**), the wild-type strain USA300 and its knock-out mutant USA300ΔPVL (**B**) and *pvl*-positive (**C**) and *pvl*-negative (**D**) clinical isolates from invasive diseases. After 1 h of incubation with bacteria the cells were washed, stained with annexin V and propidium iodide (taking another hour) and then cell death was measured by flow cytometry. The values represent the mean ± SEM of at least three independent experiments.

To investigate the impact of defined virulence factor expression we transformed *S. carnosus* TM300 with a plasmid encoding the genes for PVL, α-toxin, protein A (Spa) or PSMs, respectively (Table 1). Using live bacteria with these constructs revealed that the expression of PVL most efficiently induced neutrophils cell death (Figure 3A). The effect of TM300+PVL was comparable to the cytotoxic potential of clonally independent MRSA (ST239) and MSSA (6850) strains (Figure 3B) and of *pvl*-positive clinical isolates, which were recovered from severe invasive (including necrotizing pneumonia) diseases (Figure 3C). However, cytotoxicity was not restricted to PVL-expressing strains, as live bacteria of some *pvl*-negative isolates compromised cell viability to a similar extent (Figure 3D). Moreover, we could not detect differences between strain USA300 and the corresponding mutant USA300ΔPVL (Figure 3B), indicating that the presence of the *pvl*-gene does not necessarily contribute to neutrophils cell death following phagocytosis of bacteria. We also failed to block the cytotoxic effect of USA300 by the use of antibodies against PVL (Figure S5). These findings indicate that other staphylococcal factors can also induce cell death, which might mask the cytotoxic function of PVL. However, the expression of α-toxin and PSMs in TM300 had no effect on neutrophils. Apparently, PSMs need to accumulate to lyse neutrophils, as the corresponding bacterial supernatants, which contained PSMs, were cytolytic (Figure S6) [16].

**Table 1:** Strains used in this study.

Strain	Mutations			Description								Source
	plasmid	gene	template	a-toxin (hemolysis)			PVL				description	
							PCR		Blot			
***Escherichia coli***												
E.coli pQE30UA-lukF (USA300)	pQE30UA-lukF (USA300)	*luk*F	USA300	−			lukF				heterologous expression of PVL	this work
E.coli pQE30UA-lukS (USA300)	pQE30UA-lukS (USA300)	*luk*S	USA300	−			lukS				heterologous expression of PVL	this work
***Staphylococcus carnosus***												
TM300	−	−	−	−			−		−		WT	[29]
TM300+α-tox	pNXR100hla	*hla*	Wood46	++							heterologous expression of α-hemolysin	this work
TM300+PVL (i)	pXR100PVL	*luk*F + *luk*S	USA300	−			+		+++		heterologous expression of PVL	this work
TM300+ProtA	pNXR15spa	*spa*	CowanI	−			−				heterologous expression of protein A	this work
TM300+PSMs (i)	pXR100psm	*psm*	USA300	−			−				heterologous expression of psmA1 to psmA4	this work
TM300+FnBPA (i)	pXR100FnBPA	*fnb*A	8325-4	−			−				heterologous expression of FnBPA	[30]
***Staphylococcus aureus***												
USA300	−	−	−	++			+		+++		WT (CA-MRSA)	[12]
USA300ΔPVL	−	Δ*pvl*	−	++			−		−		PVL knock out mutant of strain USA300	[12]
ST239 (635/93)	−	−	−	++			−		−		WT (MRSA) from wound infection	(W. Witte, Wernigerode)
6850	−	−	−	++			−		−		WT (MSSA) from osteomyelitis	[31]
Cowan I	−	−	−	−			+		−		WT, high producer of protein A	ATCC 12598
DU5889 (CowanI Δspa)	−	Δ*spa*	−	−							Protein A knock out mutant of strain Cowan I	[32]
DU5889 pNXR100hla	pNXR100hla	*hla,* Δ*spa*	Wood46	+							heterologous expression of α-hemolysin	this work
Cowan I pNXR100hla	pNXR100hla	*hla,*	Wood46	+							heterologous expression of α-hemolysin	this work
***Staphylococcus aureus,*** **clinical isolates, ** ***pvl*** **+**												
F1240	−	−	−		++	+		++		WT (MSSA) from necrotizing pneumonia		this work
A1712	−	−	−		+	+		++		WT (MSSA) from necrotizing fasciitis		this work
A16040	−	−	−		++	+		++		WT (MSSA) from skin infection		this work
L2339	−	−	−		+/−	+		++		WT (MRSA) from sepsis following skin infection		this work
K4050	−	−	−		+	+		+/−		WT (MSSA) from sepsis after operation		this work
K2479	−	−	−		++	+		+		WT (MSSA) from sepsis following pneumonia		this work
***Staphylococcus aureus,*** **clinical isolates, ** ***pvl*** **-**												
K4436	−	−	−		+/−	−				WT (MRSA) from sepsis		this work
A3478	−	−	−		+	−				WT (MRSA) from wound infection		this work
A934	−	−	−		+	−				WT (MRSA) from abscess		this work
F2414	−	−	−		+/−	−				WT (MRSA) from pneumonia		this work
A18504	−	−	−		++	−				WT (MSSA) from wound infection		this work
A4206	−	−	−		+/−	−				WT (MSSA) from wound infection		this work

(i) xylose inducible, WT = wild-type isolate.

All strains were tested for hemolysis (sign for α-toxin production) on blood agar plates after 24 h and results are listed semi-quantitatively in four categories: -, no hemolysis; +/−, borderline; +, ++, efficient hemolysis. PVL production of different strains was determined by Western blot analysis of bacterial culture supernatants as described and shown in figures S1B, S1C. The results are listed semi-quantitatively in five categories: -, no PVL production; +/−, borderline; +, low; ++, +++, high amounts of PVL production.

Besides PVL, the expression of protein A moderately decreased the number of intact cells (Figure 3A). This is further demonstrated by using strain Cowan I, which is a high producer of protein A, whereas two isogenic mutants (Δ*spa*) were much less cytotoxic (Figure 3E). Although protein A is known to be a cell wall-anchored protein with an anti-phagocytic effect [14], we observed an increased rate of cell death. In our experiments, the action of protein A was dependent on the expression by bacteria, which exhibit protein A on the bacterial surface. This phenomenon was not specific for protein A, as the expression of another wall-associated protein, namely fibronectin-binding protein A (FnBPA), also reduced the number of intact neutrophils (Figure 3F). In general, phagocytosis of pathogens triggers mechanisms to kill ingested bacteria. Further on, it has been shown that phagocytosis significantly accelerates neutrophils apoptosis, which appears to contribute to the resolution of the inflammatory response [21],[22]. These processes promote healthy resolution and could be an explanation for the enhanced rate of cell death caused by bacteria holding virulent surface proteins. This assumption is further confirmed by apoptotic features detected in neutrophils (Figure S7, annexin V-positive cells).

**Figure 4:**
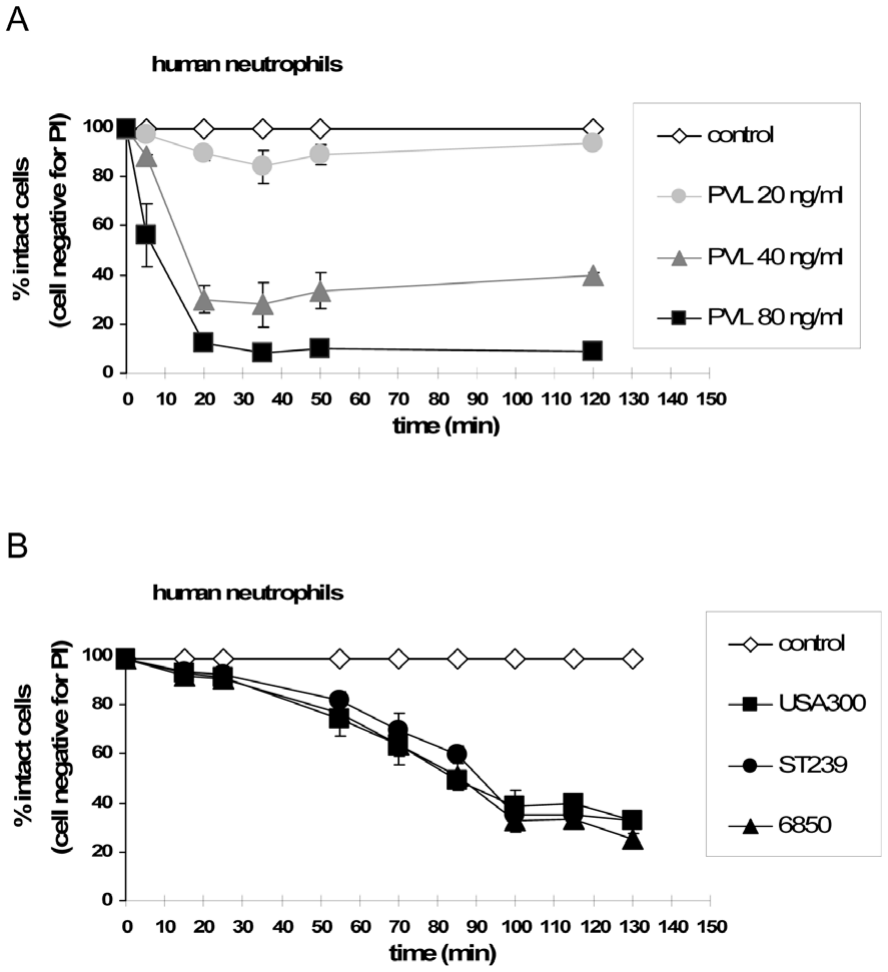
Time-dependent effect of purified PVL vs. live bacteria on neutrophil cell death induction. Human neutrophils were freshly isolated and 1×10^6^ 0.5 ml^−1^ cells were stimulated with purified PVL (**A**) or with live bacteria of wild-type strains at an MOI of 100 (**B**). Cell death was determined every 15 min. For this, cells were washed, rapidly (for 5 min) stained with PI and cell death was instantly determined by flow cytometry. The values represent the mean ± SEM of at least three independent experiments.

However, bacterial toxins, such as PVL and PSMs, could interfere with the physiological functions of neutrophils, by rapidly and prematurely killing cells. To investigate this possibility we analysed neutrophils cell death in a time-dependent manner. Challenge with PVL (≥40 ng/ml) induced cell death within the first 20 min (Figure 4A), whereas incubation of neutrophils with live bacteria resulted in a much slower rate of death induction (within 2–3 h), which is most likely associated with the neutrophils physiological function [21]. Using PVL-expressing (USA300) or non PVL-expressing (ST239, 6850) strains did not reveal any differences (Figure 4B).

### The cytotoxic effect of bacterial culture supernatants is dependent on PVL expression

**Figure 5:**
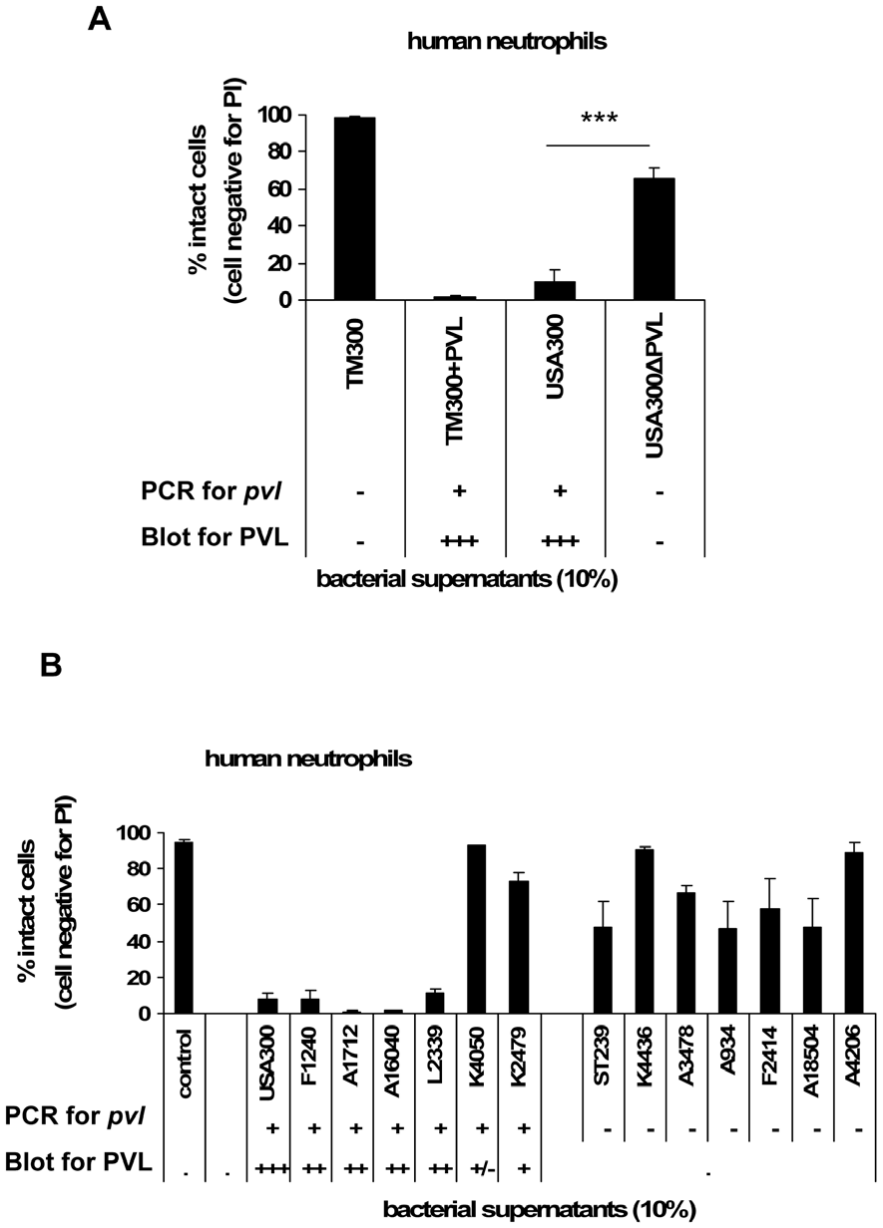
The cytotoxic effect of bacterial culture supernatants is dependent on PVL expression. Human neutrophils were freshly isolated and 1×10^6^ 0.5 ml^−1^ cells were incubated with bacterial supernatants, which were prepared from overnight cultures of different strains and used for stimulating cells (10%). In these experiments we used bacterial supernatants of the heterologous expression strain TM300+PVL and of the wild-type strain USA300 and its knock-out mutant USA300ΔPVL (**A**); furthermore we used bacterial supernatants of *pvl*-positive and *pvl*-negative clinical isolates from invasive diseases (**B**). The presence of the *pvl*-gene in the indicated strains and the amount of PVL production in the bacterial supernatants is given semi-quantitatively as listed in table 1 and demonstrated in Figure S1C. After 30 min of incubation of the cells with bacterial supernatants, cells were washed, rapidly (for 5 min) stained with PI and cell death was instantly determined by flow cytometry. The values represent the mean ± SEM of at least three independent experiments. *** P≤0.001 (independent t-test comparing the rate of intact cells after stimulation with supernatants of USA300 and USA300ΔPVL).

PVL is a bacterial exotoxin, which is rapidly released and could act on cells at the infection sites. To mimic this situation, we stimulated neutrophils with sterile-filtered bacterial supernatants from overnight cultures. Culture media from strain TM300+PVL induced rapid cell death within 20 min, whereas supernatants from the control strain TM300 did not affect cell integrity. Further on, comparing supernatants from the wild-type strain USA300 with supernatants from the corresponding knock-out mutant USA300ΔPVL revealed that culture media from the PVL-deletion strain had a much reduced ability to induce cell death, as the majority of cells remained intact (Figure 5A). The impact of PVL release was further strengthened by testing clinical isolates. Supernatants from four out of six *pvl*-positive strains, recovered from severe (including necrotizing) diseases, had a much higher cytotoxic activity than supernatants from *pvl*-negative strains (also recovered from severe invasive diseases). It is of particular importance, that PVL secretion of the strains (measured by Western blot in the bacterial supernatants; Figure S1C) clearly corresponded to the cytotoxic activity in all cases (Figure 5B).

## Discussion

The role of PVL in severe CA-MRSA infections is debated due to conflicting data from epidemiological studies, *in vitro* cell culture experiments, and different animal disease models [9],[10],[12],[23]. As PVL was found in almost all MRSA strains that cause CA-MRSA infections, such as necrotizing pneumonia, skin- and soft tissue infections, it was assumed to be a crucial virulence factor [4],[6]. These disease entities are characterized by massive tissue necrosis and leukopenia, which has been linked to the ability of PVL to kill neutrophils, the primary defending cells against invading bacteria. However, different disease models in mice, in which USA300 and the corresponding knock-out mutant were used, failed to detect a pathogenic function for PVL [10],[12]. In line with these data, we found that murine neutrophils, isolated from different commonly used mice strains, were quite insensitive to PVL. Neutrophils from Java monkeys, a species much more closely related to humans, were not affected by PVL. The reason for the differential sensitivity of cells from various species is completely unknown, but receptors/signal transduction pathways, which are confined to certain species, might be involved. By contrast, all PSM-types tested lysed neutrophils from different species equally efficient and induced membrane damaging effects [16]. Since murine and simian cells are largely resistant to PVL, PSMs might play a more dominant role in *S. aureus* infections in mice or non-human primates than in humans, especially when high *S. aureus* inocula are needed to cause diseases. Our data strongly suggest that animal models using mice or non-human primates do not correctly replicate *S. aureus* diseases in humans, at least if the role of PVL is elucidated. As neutrophils from rabbits are much more susceptible to PVL, this species is most likely more appropriate to study the function of PVL in necrotizing diseases.

Very recently, a rabbit bacteremia model has been published, which describes a modest and transient effect of PVL in the acute phase of infection [15]. However, this type of infection might not show the full pathogenicity of PVL expression, as during bacteremia staphylococci are directly exposed to cells of the immune system. In our experiments, we could not detect differences in virulence between PVL-expressing and *pvl*-negative (knock-out mutants or wild-type isolates) strains, when live bacteria were directly phagocytized by neutrophils. This is in line with other published data, demonstrating that disruption or absence of the *pvl*-gene in *S. aureus* wild-type isolates (including USA300) did not alter their capacity to induce neutrophils cell death [12],[24]. Nevertheless, as cell death of neutrophils is part of the physiological immune response following phagocytosis of bacteria [21],[22] and as *S. aureus* wild-type isolates express a wide variety of factors promoting this process (e.g. diverse surface proteins) [1], the effect of secreted PVL on human neutrophils might be masked in this model. Furthermore, it is reasonable to suspect that PVL is not (highly) expressed, when staphylococci are instantly phagocytized by neutrophils, as toxic virulence factors were found to be down-regulated after internalization of bacteria [25]. Like other toxins, PVL is mainly expressed in the post-exponential bacterial growth phase [26], which is most likely reached in encapsulated infection foci, e.g. folliculitis, abscesses, tissue necrosis. Only recently, high expression of PVL was found directly in clinical samples from cutaneous abscesses of invasive CA-MRSA infections [27]. Here, PVL most likely accumulates and can also exert systemic pathogenic actions upon entering the bloodstream. In human neutrophils, low doses of PVL were sufficient to cause cell death, which correspond to amounts produced by clinical CA-MRSA strains [28]. Granted that the action of PVL involves yet unknown host receptors/signal transduction pathways, PVL might interfere with various functions of susceptible cells. Furthermore, it is reasonable to speculate that host organisms can become even more vulnerable against PVL, e.g. following an infection with influenza virus. Additional studies on human cells and in susceptible animal models (rabbits) will be necessary to clarify these possibilities and to better define the functions of PVL in staphylococcal infections.

Taken together, our results clearly demonstrate that PVL is a strong cytotoxic factor for human neutrophils, which can play an important role in CA-MRSA infections. Our results do not contradict previously published work, as we could not find an effect of PVL on murine neutrophils or when bacteria were directly phagocytised by neutrophils. However, under certain pathogenic conditions, such as necrosis and abscesses, which are characteristic for severe invasive *S. aureus* diseases, PVL could exert its function as a cytotoxic exotoxin in susceptible organisms. The premature cell death of neutrophils may be extremely relevant in the virulence of CA-MRSA. As neutrophils are the major defense against invading bacteria, their excessive cell death most likely largely compromises the host's immune system. Furthermore, uncontrolled neutrophils cell damage discharges many pro-inflammatory components within the host tissue, which could also essentially promote disease development. These results are important for ongoing efforts to find therapeutics against *S. aureus* infections. Due to the rapid spread of CA-MRSA strains and situations, which favour *S. aureus* infections at a large scale, e.g. epidemic of influenza, there is an urgent need for efficient preventive and therapeutic strategies.

## Materials and Methods

### Ethics statement

Taking of blood samples from humans and animals and cell isolation were conducted with approval of the local ethics committee (Ethik-Komission der Ärztekammer Westfalen-Lippe und der Medizinischen Fakultät der Westfälischen Wilhelms-Universität Münster). Human blood samples were taken from healthy blood donors, who provided written informed consent for the collection of samples and subsequent neutrophil isolation and analysis. All animals were handled in strict accordance with good animal practice and animal keeping and taking of blood samples were supervised by the veterinary office of Münster (Veterinäramt der Stadt Münster).

### Bacterial strains and cultures

Bacterial strains used in this study are listed in table 1. They were all characterized for presence of genes encoding PVL and α-toxin by PCR. Gene expression was investigated by Western blots for PVL (Figure S1B+C) or by hemolysis on sheep blood agar plates (sign for α-toxin production). For cell culture and animal experiments with live staphylococci, bacteria were grown overnight at 37°C in Müller-Hinton medium (MH, containing antibiotics/xylose, if mutants are used) without shaking. Bacteria were washed in PBS and resuspended in PBS with 1% HSA. Neutrophils were incubated with bacterial suspensions, resulting in a multiplicity of infection (MOI) as indicated. Bacterial supernatants were prepared by growing bacteria in 5 ml of brain-heart infusion (BHI) broth (Merck) in a rotary shaker (160 rpm) at 37°C for 12–14 h and pelleted for 10 min at 3350 g. Supernatants were sterile-filtered through a Millex-GP filter unit (0.22 µm; Millipore) and used for the experiments. For PVL isolation, *E. coli* TG1 strains containing expression vectors for lukF-PV and lukS-PV were grown in Luria Bertani (LB)-media with IPTG (1 mM) and ampicillin (100 mg/ml) and cell lysates were used to purify PVL (Figure S1A).

### Plasmid construction and transformation

Different genes were amplified by PCR using chromosomal DNA from different strains (Table S1) as template. To create *S. carnosus* strains, which express virulence factors of *S. aureus,* we used two basic vectors, the xylose inducible pXR100 and the pNXR100, which is a non-inducible derivate of the pXR100. For the expression of lukF-PV and lukS-PV in *E. coli* TG1 the commercial IPTG inducible pQE30UA was used. For creation of the expression vectors the respective genes were amplified by PCR, purified and digested. The basic vectors were also digested corresponding to the genes. After ligation *S. carnosus* TM300 and *E. coli* were transformed by protoplast transformation or CaCl–method.

### Generation of purified staphylococcal virulence factors and antibodies

The Six-histagged lukF-PV and lukS-PV proteins from *E. coli* were purified by nickel-nitrilotriacetic acid affinity resin (Qiagen, Germany). α-toxin and Protein A (P3838) were obtained from Sigma-Aldrich Chemie GmbH (Germany). PSMα1 – PSMα3 were synthesized by Genosphere Biotechnology (France). Polyclonal antibodies against lukF-PV and lukS-PV were raised separately and together in rabbits by standard procedures and this was performed by Genosphere-Biotechnology (France).

### Preparation and culture of neutrophils

Human, rabbit and Java monkey polymorphonuclear cells (neutrophils) were freshly isolated from Na citrate-treated blood of healthy donors. Neutrophils from BALB/c and C57/BL6 mice were prepared from bone marrow. For neutrophil-isolation, dextran-sedimentation and density gradient centrifugation using Ficoll-Paque Plus (Amersham Bioscience) was used according to the manufacturer's instruction. Cell purity was determined by Giemsa staining and was always above 99%. For murine cells, sedimented cells were used as neutrophils and, in addition, were further deprived of CD3^+^ (T cells), CD19^+^ (B cells), and CD11c^+^ (dendritic cells) cells using MACS technology (Miltenyi Biotech, Bergisch- Gladbach) according to the manufacturer’s instruction. Resulting cells were <0.1% CD3^+^, CD19^+^, or CD11c^+^ and <95% CD11b^+^ and Gr1^+^. Neutrophils were resuspended at a final density of 1×10^6^ cells/0.5 ml in RPMI 1640 culture medium (PAA Laboratories GmbH) supplemented with 10% heat-inactivated FCS (PAA Laboratories GmbH) and immediately used for the experiments. All incubations were performed at 37°C in humidified air with 5% CO_2_.

### Cell culture experiments and measurements of oxidative burst activity and cell death

All experiments were performed in 24-well plates and neutrophils were incubated with PVL, α-toxin, PSMs, live bacteria or bacterial supernatants at the indicated concentrations. Oxidative burst activity was determined after 10 min of incubation using a phagoburst test (Orpegen Pharma) according to the manufacturer's instruction. Measurement of cell death was performed after 1 h of incubation followed by washing and double staining of cells with annexin V-FITC and propidium iodide (PI) (taking 1 hour) and then cells were analyzed in a FACScalibur flow cytometer using an annexin V-FITC apoptosis detection kit (Becton Dickinson). For analysis of time-dependent cell death inductions, cells were incubated for the indicated time periods, followed by washing and single staining with PI (taking 10 min) and then cells were immediately analysed by flow cytometry.

### Light and transmission electron microscopy

A live cell imaging system (Zeiss) was used to obtain light micrographs. For transmission electron microscopy, 5×10^6^/2.5 ml neutrophils were incubated with PVL at the indicated concentrations for 1 h. Then the cells were washed three times with PBS, fixed in 3% glutaraldehyde, stained in 1% osmium tetroxide and embedded in epoxy resin in the culture dish *in situ*. Electron micrographs were obtained using imaging plate technology.

### Statistical analysis

Unpaired Student’s *t*-test was performed to compare cell survival. A value of P≤0.05 was considered significant in all cases.

## Supporting Information

Table S1 Vector construction
(0.08 MB PDF)

Figure S1 Western-blot and SDS-PAGE analysis of *S. aureus* USA300 lukF-PV and lukS-PV and of PVL in bacterial supernatants of indicated strains. His-Tag lukF and lukS proteins were expressed in *E. coli* using pQE30UA and proteins were purified on NI-NTA resin. After separation by SDS-PAGE, proteins were visualized by Coomassie blue. For Western-blot analysis, proteins separated on SDS-page were blotted onto a nitrocellulose membrane. Detection of PVL (lukF and lukS) was done with anti-PVL antibodies raised in rabbits followed by incubation with anti-rabbit alkaline phosphatase conjugated antibodies and bands were visualized in a color reaction using avidin alkaline phosphatase. Molecular weight standards are in kDa (Figure S1A). To detect PVL released in bacterial culture supernatants, staphylococcal strains were grown in 5 ml of brain-heart infusion (BHI), supernatants were sterile-filtered as described and were used for Western-blot analysis (Figures S1B, S1C). The amount of PVL was determined semi-quantitatively in five categories: -, no PVL production; +/-, borderline; +, low; ++, +++, high and very high PVL production. The results are also listed in Table 1 and Figure 5.
(1.27 MB TIF)

Figure S2 Cell death induced by PVL in neutrophils lacks apoptotic features. Human neutrophils were freshly isolated and 1×10^6^ 0.5 ml^−1^ cells were incubated with increasing doses of purified PVL with or without zVAD-fmk (50 μM) as indicated. zVAD is a pan-caspase inhibitor (Enzyme Systems), which inhibited apoptotic cell death induced by α-toxin in mononuclear cells [33]. After 1 h cells were double-stained with propidium iodide to detect necrosis-like membrane damage and with annexin V-fluorescein isothiocyanate to detect apoptotic phosphatidylserine exposure to the cell surface by flow cytometry. Figure S2A shows the percentage of intact cells and the values represent the mean ± SEM of four different experiments. No significant differences were detected in cells treated with zVAD compared to cells treated without zVAD. Figure S2B shows one representative flow cytometric measurement. We could not detect annexin V positive cells at any dose of PVL tested. These results indicate that rapid cell death induced by PVL lacks apoptotic features and is most likely due to necrosis.
(2.01 MB TIF)

Figure S3 Murine neutrophils are largely resistant to PVL irrespective of their maturation and inflammatory state. In Figure S3A neutrophils from BALB/c mice were isolated from bone marrow or were analysed by flow cytometry (gating) in whole peripheral blood, as indicated. Cells were stimulated with lukS-PV or with lukF-PV or with both components (PVL: 4 µg/ml) for 90 min. After stimulation cells were stained with annexin V and propidium iodide and then the rate of cell death was measured by flow cytometry. In Figure S3B neutrophils from BALB/c mice (control mice, stess-induced mice or *S. aureus*-infected mice) were isolated from bone marrow. For stress-induction mice were fixed (immobilized) for 30 min/day on 4 consecutive days. It has been shown that fixation leads to stress induction that can be measured by increased levels of glucocorticoids in the serum [34]. For *S. aureus* infection mice were infected with *S. aureus* SH1000 (2×10^7^ bacteria) into the footpad 7 days before cell isolation. For the experiments, 1×10^6^ cells were stimulated with PVL (4 µg/ml) for 90 min. After stimulation cells were stained with annexin V and propidium iodide and then the rate of cell death was measured by flow cytometry.
(1.19 MB TIF)

Figure S4 Low doses of PVL induce proinflammatory activation of human neutrophils. Human neutrophils were freshly isolated and 1×10^6^cells were stimulated with different doses of PVL (4 - 400 ng/ml) for 60 min. After stimulation RNA was isolated from the cells and expression of selected genes was confirmed by real-time reverse transcription-polymerase chain reaction (RT-PCR). The primers used for PCR analysis were as follows: *IL-1β* forward, 5′-GCGGCCAGGATATAACTGACTTC-3′; *IL-1β* reverse, 5′-GCGGCCAGGATATAACTGACTTC-3′-TCCACATTCAGCACAGGACTCTC-3′-GCGGCCAGGATATAACTGACTTC-3′; *IL-8* forward, 5′-GCGGCCAGGATATAACTGACTTC-3′-CTTGTTCCACTGTGCCTTGGTT-3′-GCGGCCAGGATATAACTGACTTC-3′; *IL-8* reverse, 5′-GCGGCCAGGATATAACTGACTTC-3′-GCTTCCACATGTCCTCACAACAT-3′-GCGGCCAGGATATAACTGACTTC-3′; *GAPDH* forward, 5′-GCGGCCAGGATATAACTGACTTC-3′-TGCACCACCAACTG CTTAGC-3′-GCGGCCAGGATATAACTGACTTC-3′ ; *GAPDH* reverse, 5′-GCGGCCAGGATATAACTGACTTC-3′-GGCATGGACTGTGGTCATGAG-3′-GCGGCCAGGATATAACTGACTTC-3′; *RPL* forward, 5′-GCGGCCAGGATATAACTGACTTC-3′-AGGT ATGCTGCCCCACAAAAC-3′-GCGGCCAGGATATAACTGACTTC-3′; *RPL* reverse, 5′-GCGGCCAGGATATAACTGACTTC-3′-TGTAGGCTTCAGACGCACGAC-3′-GCGGCCAGGATATAACTGACTTC-3′. The relative expression was calculated as 2^ΔC^t^specific gene^ / 2^ΔC^t^mean (houskeeping gene)^, using glyceraldehyde phosphate dehydrogenase (*GAPDH*) and ribosomal protein L13a (*RPL*), as endogenous housekeeping control genes (A and B). The culture supernatants of the stimulated cells were collected after 6 h and 16 h and the protein levels of IL-1β were determined by ELISA (Becton Dickinson) according to the manufacturer's instructions (C). The values represent the means ±SD of three independent experiments. Similar experiments were performed with murine neutrophils from BALB/c mice, but here no induction of murine chemokine KC expression could be detected (data not shown).
(0.64 MB TIF)

Figure S5 Antibodies against PVL cannot prevent the cytotoxic effect of the PVL-expressing strain USA300. Human neutrophils were freshly isolated and 1×10^6^ 0.5 ml^−1^ cells were incubated with purified PVL (80 ng/ml), with live bacteria (MOI 50) of the PVL-expressing strain USA300 or of the wild-type isolate ST239, which lacks the gene for PVL. In bacterial supernatants protein A was removed to avoid unspecific binding of antibodies to protein A. Antibodies against PVL (15 µg/ml) were added to the cells before cells were incubated with PVL, live bacteria. Co-incubation with antibodies completely prevented the effect of purified PVL, whereas control antibodies (against extracellular matrix protein Emp) had no effect. The effect of strain USA300 was not affected by the addition of antibodies against PVL and was similar to the action of wild-type strain ST 239. These results suggest that *S. aureus* wild-type isolates express a multitude of virulence factors, which promote cell death induction. However, as cell death of neutrophils is part of the immediate immune response following exposure to pathogens and/or phagocytosis of bacteria, the action of secreted PVL from USA300 might be masked in this model. *** P≤0.001 comparing the rate of intact cells between control and stimulated cells.
(0.08 MB TIF)

Figure S6 Bacterial supernatants from TM300+PSMs induce neutrophil lysis. Human neutrophils were freshly isolated and 1×10^6^ 0.5 ml^−1^ cells were incubated with bacterial supernatants of the strains TM300 and TM300+PSMs. Bacterial supernatants were prepared from bacteria grown in brain-heart infusion broth in a rotatory shaker for 40 h and supernatants were sterile filtered and added to the cell culture medium at a final concentration of 30%. The values represent the mean ± SEM of three independent experiments. ** P≤0.01 comparing the rate of intact cells between control and stimulated cells. Supernatants from the parent strain TM300 did not affect cell viability, whereas supernatants from strain TM300+PSMs induced cell lysis. As live bacteria from TM300+PSMs did not induce cell death (Figure 3A), these results indicate that PSMs have to accumulate in the bacterial supernatants to reach sufficient high concentrations to induce cell lysis.
(0.07 MB TIF)

Figure S7 *S. carnosus* TM300, which heterologously expresses covalently bound surface proteins, induce apoptotic cell death in human neutrophils. Human neutrophils were freshly isolated and 1×10^6^ 0.5 ml^−1^ cells were incubated with live bacteria of *S. carnosus* strains expressing protein A or FnBPs at an MOI of 200. After 1 h of incubation cells were washed, stained with annexin V and propidium iodide (taking another hour) and then cell death was measured by flow cytometry. This figure shows one representative flow cytometric measurement. Here, we could detect a clear shift towards annexin V positive cells (positive for annexin V and negative for PI: sign for early apoptosis) resulting in 15–20% apoptotic cells. By contrast, stimulation with PVL did not cause apoptotic features (see Figure S2). These results indicate that strains, which express virulent surface proteins, can induce forms of programmed cell death.
(1.79 MB TIF)
