# Supplementary material for: Staphylococcus aureus Panton-Valentine Leukocidin Is a Very Potent Cytotoxic Factor for Human Neutrophils
Source: PLoS Pathog. 2010 Jan 8;6(1):e1000715. doi: 10.1371/journal.ppat.1000715 (PMC2798753; doi:10.1371/journal.ppat.1000715)
Supplement: Table S1 — Vector construction (0.08 MB PDF) [file ppat.1000715.s001.pdf]

Table S1: vector construction

vector	basic vector	gen	primer
pNXR100	pXR100	-	ATATCTCGAGTAATTTGGACAGGGGAAGTTCGGTTA
			ATATCTCGAGCTACTTTATTGGGTGGCACTTTAGCA
pXR100PVL	pXR100	*luk*F+*luk*S	CTCGGATCCTTGTTTGGTAATGAACGGGTTTTTTCG
			CTCGGATCCAGTGAATGCCCTTATTTAAATAATCCGCCC
pNXR100spa	pNXR100	*spa*	ATATGGATCCTACATACAGGGGGTATTAATTTG
			ATATGAATTCTAAGTAAAATAAAAAATGCACTGAGC
pNXR100hla	pNXR100	*hla*	ATGGATCCATAGAAGGATGATGAAAATGAAAACAC
			ATATGAATTCGTCAGGAAAGCAAATCAAGTACATAGT
pQE30UA-lukF	pQE30UA	*luk*F	GCTCAACATATCACACCTGTAAGTGAG
			TTAGCTCATAGGATTTTTTTCCTTAGATTGAG
pQE30UA-lukS	pQE30UA	*luk*S	GATAACAATATTGAGAATATTGGTGAT
			TTATCAATTATGTCCTTTCACTTTAATTTCATG
pXR100psm	pXR100	*psm*	CTCGGATCCAACTAGACACTGCATCACGGTACGTTGATATACC
			CTCCTCGTCGACAACGCGTCAGACTACAAAGATCACGAATATTCC

Expression vectors, genes and primers. Creation of primers was done with Clone Manager (Scientific and Educational Software). The Primers were produced by Eurofins MWG Operon. NCBI accession no. of the genes: psm: BK006301; lukF und lukS: X72700; spa: M18264; hla : X01645

We applied two basic vectors, the xylose inducible pXR100 (1) and the pNXR100, witch is a non-inducible derivate of the pXR100. Transformation was performed by protoplast transformation or CaCl–methode as described previously (2;3).

References:

(1) Sandgathe A, Tippe D, Dilsen S, Meens J, Halfar M et al. (2003) Production of a human calcitonin precursor with *Staphylococcus aureus* seceretory expression and single-step recovery by expanded bed adsorption. Process Biochemistry 38: 1351-63.

(2) Grundmeier M, Hussain M, Becker P, Heilmann C, Peters G et al. (2004) Truncation of fibronectin-binding proteins in *Staphylococcus aureus* strain Newman leads to deficient adherence and host cell invasion due to loss of the cell wall anchor function. Infect Immun 72: 7155-63.

(3) Sambrook J, Maiatis T, Frisch EF, (1989). Molecular cloning: A laboratory manual. New York: Cold Spring Harbour Labotatory.
