# Supplementary figures and images for: Staphylococcus aureus Panton-Valentine Leukocidin Is a Very Potent Cytotoxic Factor for Human Neutrophils
Source: PLoS Pathog. 2010 Jan 8;6(1):e1000715. doi: 10.1371/journal.ppat.1000715 (PMC2798753; doi:10.1371/journal.ppat.1000715)

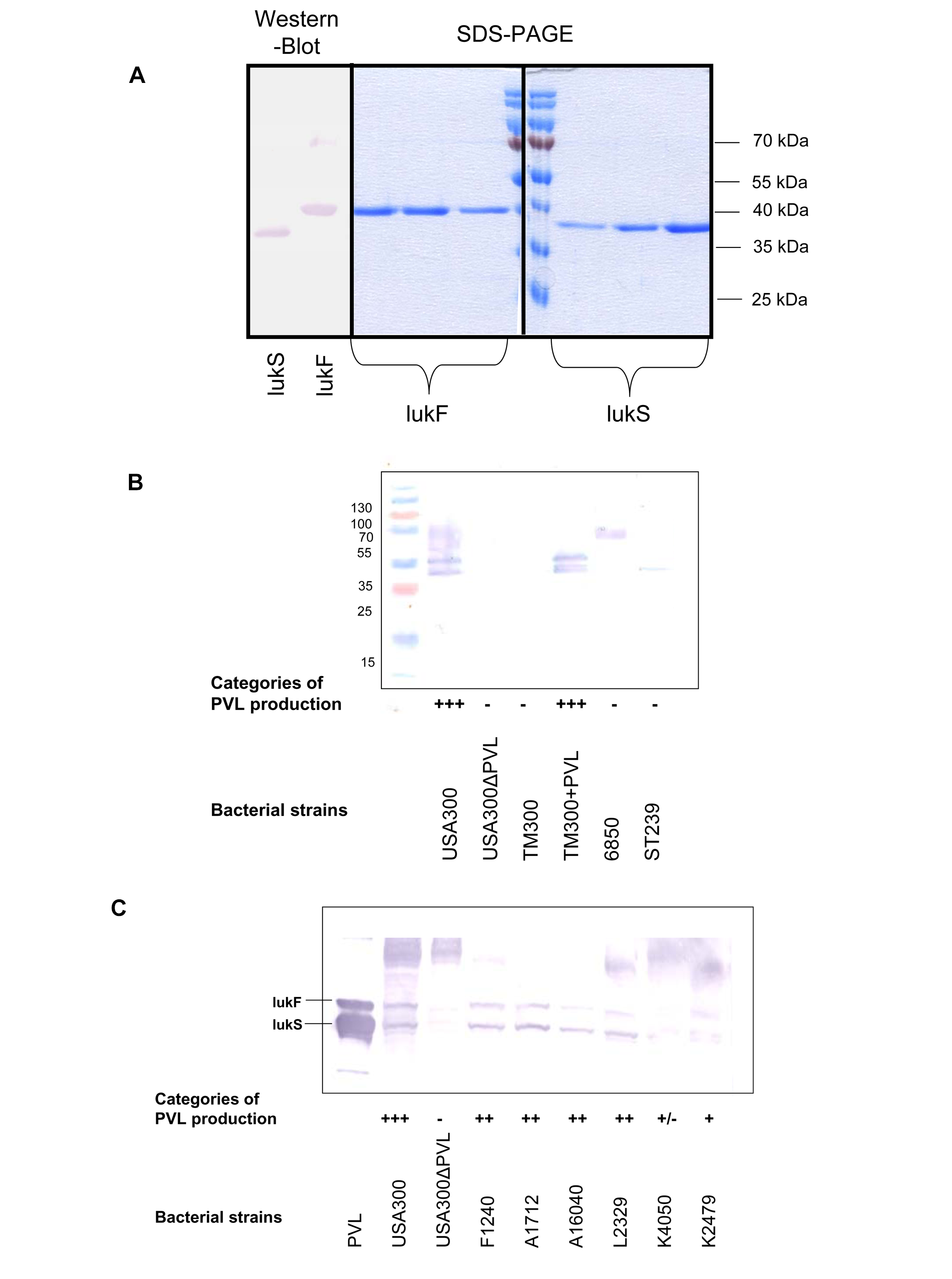

Supplement: Figure S1 — Western-blot and SDS-PAGE analysis of S. aureus USA300 lukF-PV and lukS-PV and of PVL in bacterial supernatants of indicated strains. His-Tag lukF and lukS proteins were expressed in E. coli using pQE30UA and proteins were purified on NI-NTA resin. After separation by SDS-PAGE, proteins were visualized by Coomassie blue. For Western-blot analysis, proteins separated on SDS-page were blotted onto a nitrocellulose membrane. Detection of PVL (lukF and lukS) was done with anti-PVL antibodies raised in rabbits followed by incubation with anti-rabbit alkaline phosphatase conjugated antibodies and bands were visualized in a color reaction using avidin alkaline phosphatase. Molecular weight standards are in kDa (Figure S1A). To detect PVL released in bacterial culture supernatants, staphylococcal strains were grown in 5 ml of brain-heart infusion (BHI), supernatants were sterile-filtered as described and were used for Western-blot analysis (Figures S1B, S1C). The amount of PVL was determined semi-quantitatively in five categories: -, no PVL production; +/-, borderline; +, low; ++, +++, high and very high PVL production. The results are also listed in Table 1 and Figure 5. (1.27 MB TIF) [file ppat.1000715.s002.tif]

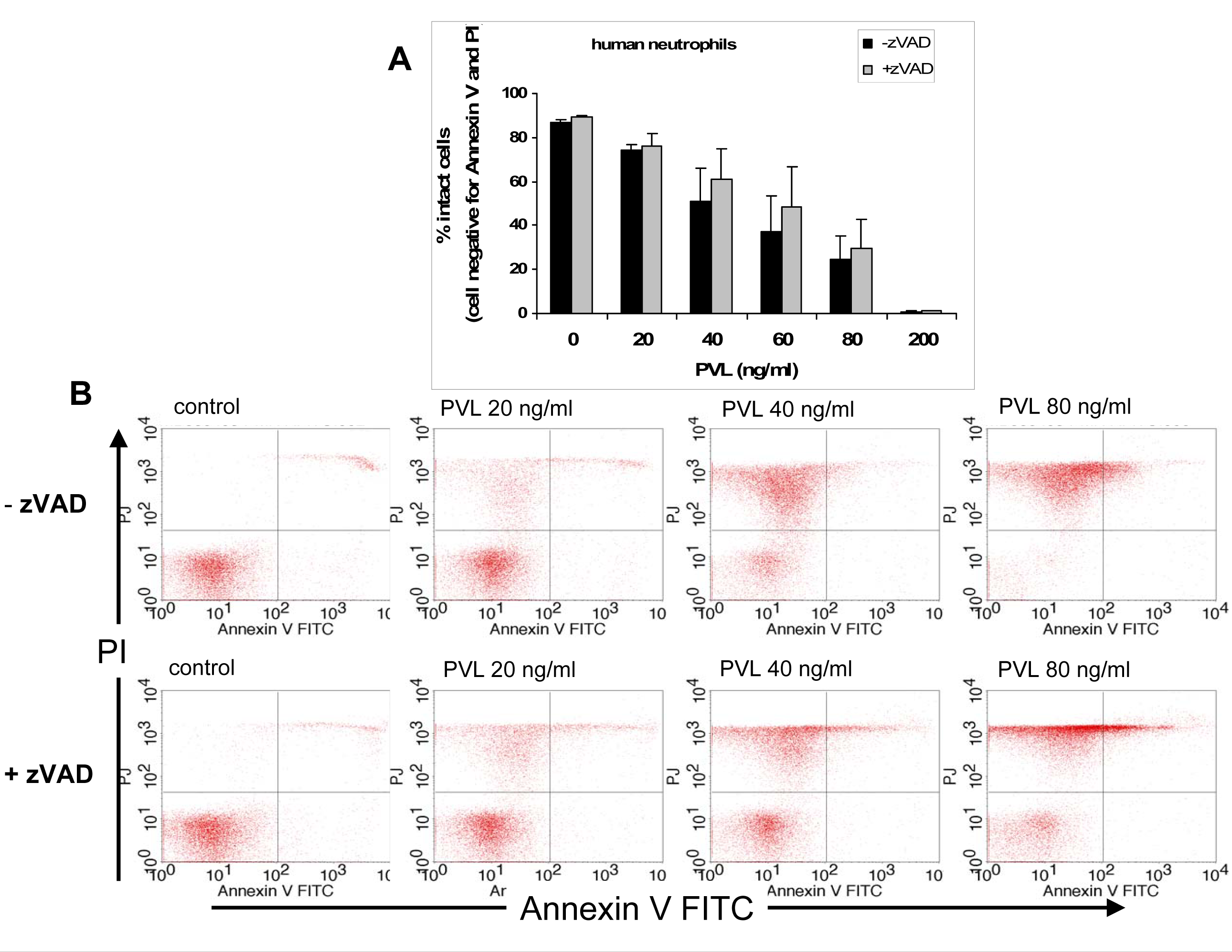

Supplement: Figure S2 — Cell death induced by PVL in neutrophils lacks apoptotic features. Human neutrophils were freshly isolated and 1×10^6^ 0.5 ml−1 cells were incubated with increasing doses of purified PVL with or without zVAD-fmk (50 μM) as indicated. zVAD is a pan-caspase inhibitor (Enzyme Systems), which inhibited apoptotic cell death induced by α-toxin in mononuclear cells [33]. After 1 h cells were double-stained with propidium iodide to detect necrosis-like membrane damage and with annexin V-fluorescein isothiocyanate to detect apoptotic phosphatidylserine exposure to the cell surface by flow cytometry. Figure S2A shows the percentage of intact cells and the values represent the mean ± SEM of four different experiments. No significant differences were detected in cells treated with zVAD compared to cells treated without zVAD. Figure S2B shows one representative flow cytometric measurement. We could not detect annexin V positive cells at any dose of PVL tested. These results indicate that rapid cell death induced by PVL lacks apoptotic features and is most likely due to necrosis. (2.01 MB TIF) [file ppat.1000715.s003.tif]

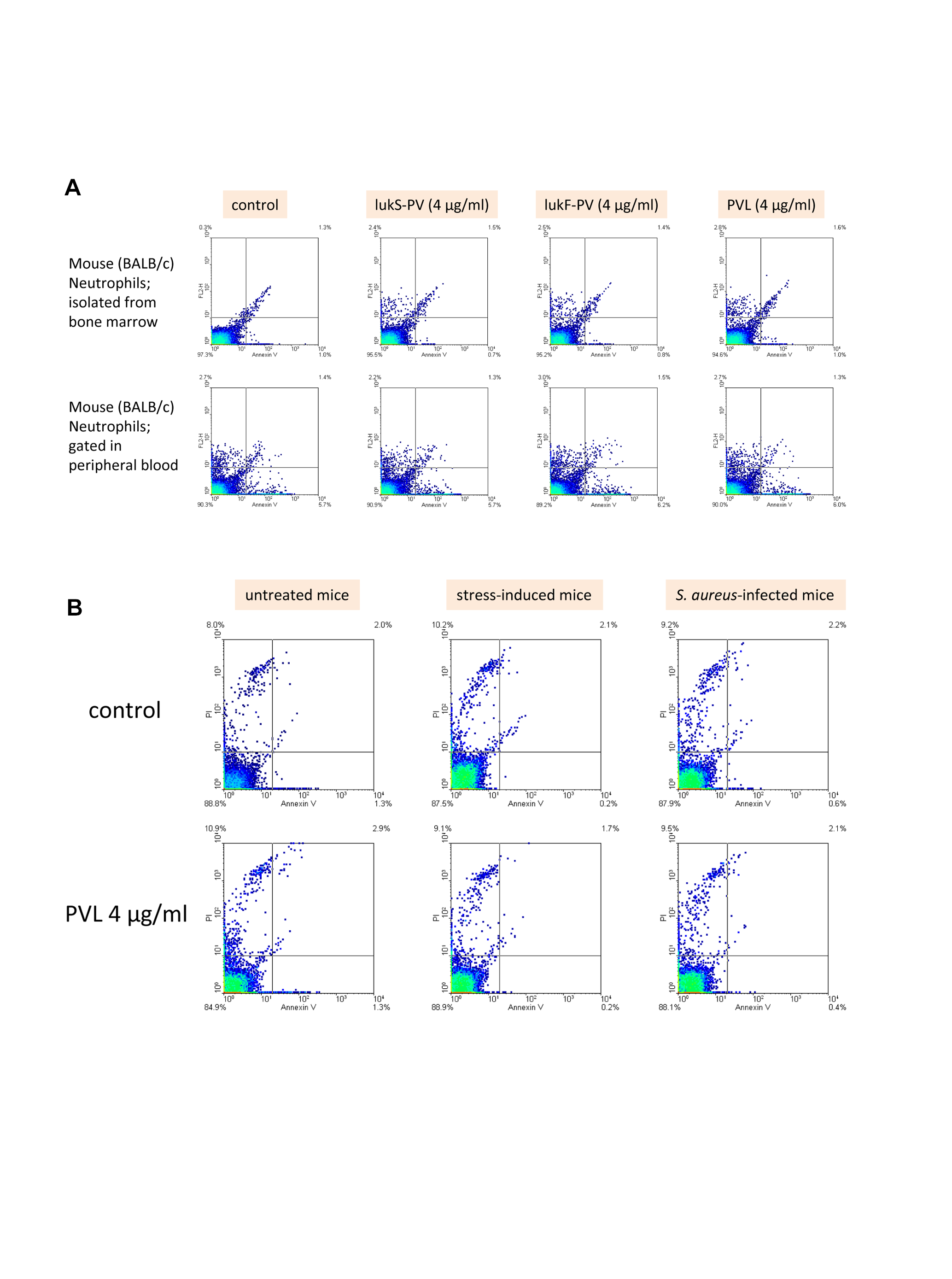

Supplement: Figure S3 — Murine neutrophils are largely resistant to PVL irrespective of their maturation and inflammatory state. In Figure S3A neutrophils from BALB/c mice were isolated from bone marrow or were analysed by flow cytometry (gating) in whole peripheral blood, as indicated. Cells were stimulated with lukS-PV or with lukF-PV or with both components (PVL: 4 µg/ml) for 90 min. After stimulation cells were stained with annexin V and propidium iodide and then the rate of cell death was measured by flow cytometry. In Figure S3B neutrophils from BALB/c mice (control mice, stess-induced mice or S. aureus-infected mice) were isolated from bone marrow. For stress-induction mice were fixed (immobilized) for 30 min/day on 4 consecutive days. It has been shown that fixation leads to stress induction that can be measured by increased levels of glucocorticoids in the serum [34]. For S. aureus infection mice were infected with S. aureus SH1000 (2×10^7^ bacteria) into the footpad 7 days before cell isolation. For the experiments, 1×10^6^ cells were stimulated with PVL (4 µg/ml) for 90 min. After stimulation cells were stained with annexin V and propidium iodide and then the rate of cell death was measured by flow cytometry. (1.19 MB TIF) [file ppat.1000715.s004.tif]

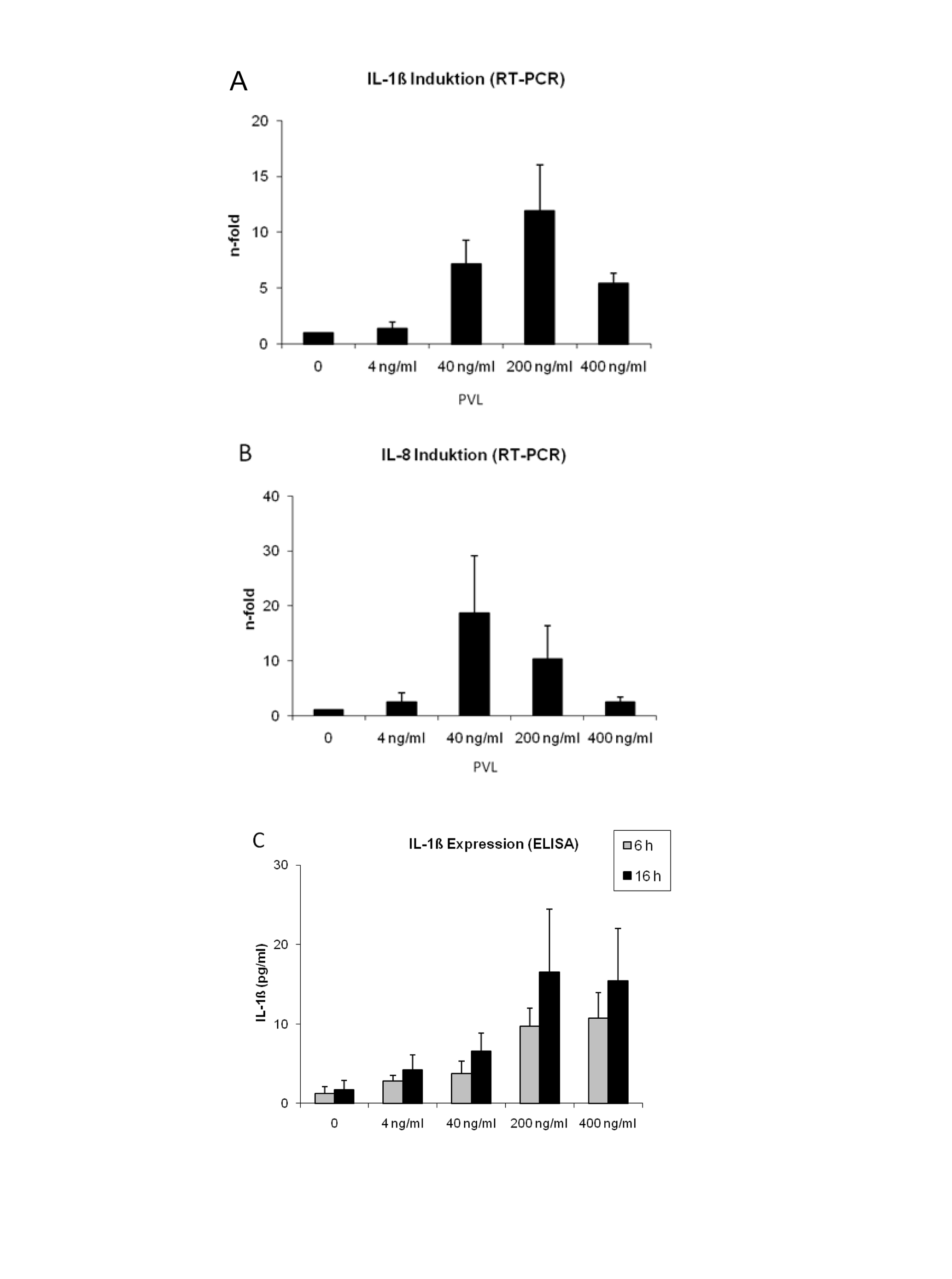

Supplement: Figure S4 — Low doses of PVL induce proinflammatory activation of human neutrophils. Human neutrophils were freshly isolated and 1×10^6^cells were stimulated with different doses of PVL (4 - 400 ng/ml) for 60 min. After stimulation RNA was isolated from the cells and expression of selected genes was confirmed by real-time reverse transcription-polymerase chain reaction (RT-PCR). The primers used for PCR analysis were as follows: IL-1β forward, 5′-GCGGCCAGGATATAACTGACTTC-3′; IL-1β reverse, 5′-GCGGCCAGGATATAACTGACTTC-3′-TCCACATTCAGCACAGGACTCTC-3′-GCGGCCAGGATATAACTGACTTC-3′; IL-8 forward, 5′-GCGGCCAGGATATAACTGACTTC-3′-CTTGTTCCACTGTGCCTTGGTT-3′-GCGGCCAGGATATAACTGACTTC-3′; IL-8 reverse, 5′-GCGGCCAGGATATAACTGACTTC-3′-GCTTCCACATGTCCTCACAACAT-3′-GCGGCCAGGATATAACTGACTTC-3′; GAPDH forward, 5′-GCGGCCAGGATATAACTGACTTC-3′-TGCACCACCAACTG CTTAGC-3′-GCGGCCAGGATATAACTGACTTC-3′ ; GAPDH reverse, 5′-GCGGCCAGGATATAACTGACTTC-3′-GGCATGGACTGTGGTCATGAG-3′-GCGGCCAGGATATAACTGACTTC-3′; RPL forward, 5′-GCGGCCAGGATATAACTGACTTC-3′-AGGT ATGCTGCCCCACAAAAC-3′-GCGGCCAGGATATAACTGACTTC-3′; RPL reverse, 5′-GCGGCCAGGATATAACTGACTTC-3′-TGTAGGCTTCAGACGCACGAC-3′-GCGGCCAGGATATAACTGACTTC-3′. The relative expression was calculated as 2ΔCtspecific gene / 2ΔCtmean (houskeeping gene), using glyceraldehyde phosphate dehydrogenase (GAPDH) and ribosomal protein L13a (RPL), as endogenous housekeeping control genes (A and B). The culture supernatants of the stimulated cells were collected after 6 h and 16 h and the protein levels of IL-1β were determined by ELISA (Becton Dickinson) according to the manufacturer's instructions (C). The values represent the means ±SD of three independent experiments. Similar experiments were performed with murine neutrophils from BALB/c mice, but here no induction of murine chemokine KC expression could be detected (data not shown). (0.64 MB TIF) [file ppat.1000715.s005.tif]

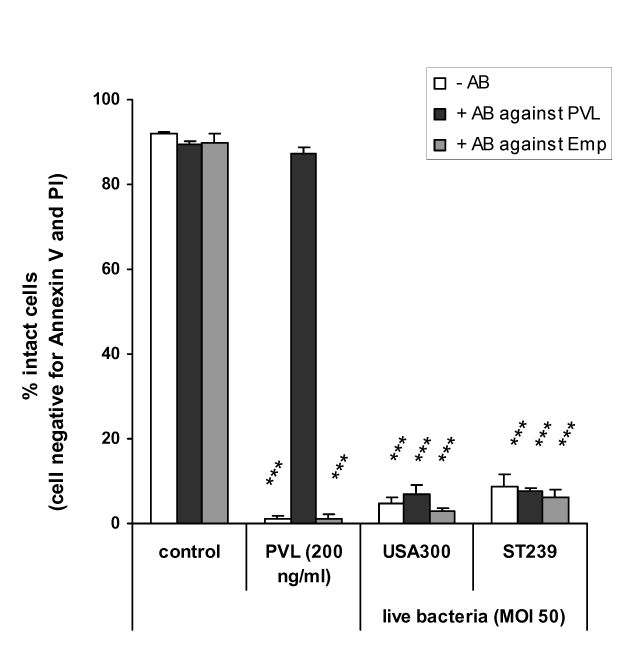

Supplement: Figure S5 — Antibodies against PVL cannot prevent the cytotoxic effect of the PVL-expressing strain USA300. Human neutrophils were freshly isolated and 1×10^6^ 0.5 ml−1 cells were incubated with purified PVL (80 ng/ml), with live bacteria (MOI 50) of the PVL-expressing strain USA300 or of the wild-type isolate ST239, which lacks the gene for PVL. In bacterial supernatants protein A was removed to avoid unspecific binding of antibodies to protein A. Antibodies against PVL (15 µg/ml) were added to the cells before cells were incubated with PVL, live bacteria. Co-incubation with antibodies completely prevented the effect of purified PVL, whereas control antibodies (against extracellular matrix protein Emp) had no effect. The effect of strain USA300 was not affected by the addition of antibodies against PVL and was similar to the action of wild-type strain ST 239. These results suggest that S. aureus wild-type isolates express a multitude of virulence factors, which promote cell death induction. However, as cell death of neutrophils is part of the immediate immune response following exposure to pathogens and/or phagocytosis of bacteria, the action of secreted PVL from USA300 might be masked in this model. *** P≤0.001 comparing the rate of intact cells between control and stimulated cells. (0.08 MB TIF) [file ppat.1000715.s006.tif]

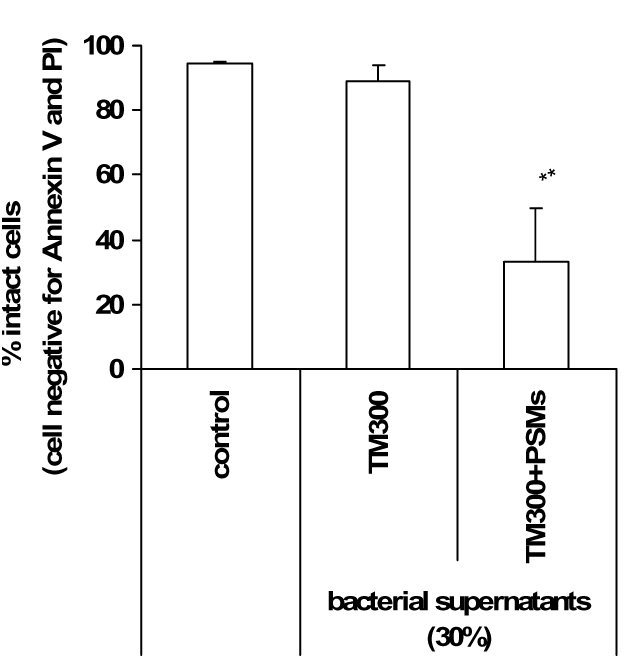

Supplement: Figure S6 — Bacterial supernatants from TM300+PSMs induce neutrophil lysis. Human neutrophils were freshly isolated and 1×10^6^ 0.5 ml−1 cells were incubated with bacterial supernatants of the strains TM300 and TM300+PSMs. Bacterial supernatants were prepared from bacteria grown in brain-heart infusion broth in a rotatory shaker for 40 h and supernatants were sterile filtered and added to the cell culture medium at a final concentration of 30%. The values represent the mean ± SEM of three independent experiments. ** P≤0.01 comparing the rate of intact cells between control and stimulated cells. Supernatants from the parent strain TM300 did not affect cell viability, whereas supernatants from strain TM300+PSMs induced cell lysis. As live bacteria from TM300+PSMs did not induce cell death (Figure 3A), these results indicate that PSMs have to accumulate in the bacterial supernatants to reach sufficient high concentrations to induce cell lysis. (0.07 MB TIF) [file ppat.1000715.s007.tif]

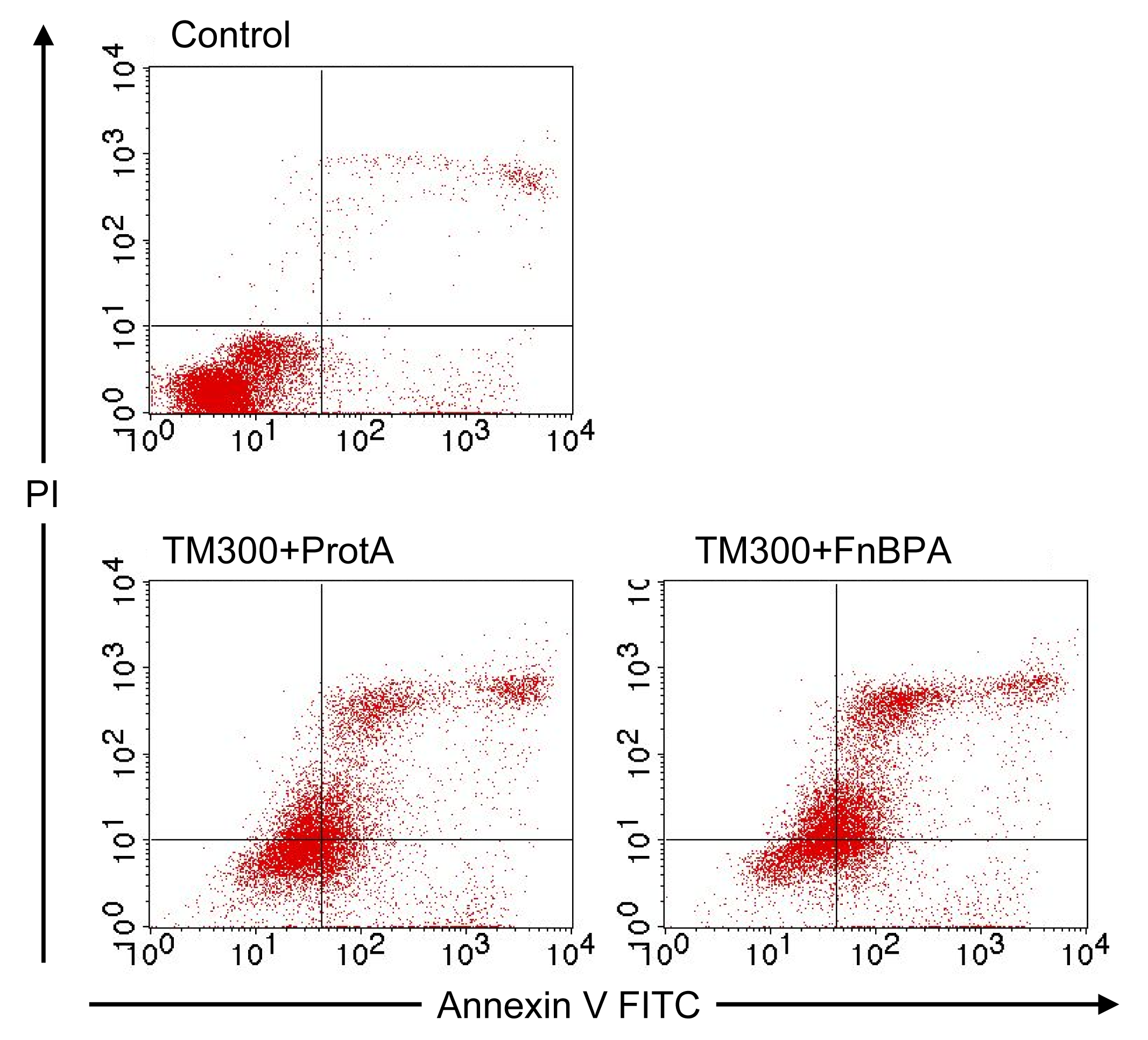

Supplement: Figure S7 — S. carnosus TM300, which heterologously expresses covalently bound surface proteins, induce apoptotic cell death in human neutrophils. Human neutrophils were freshly isolated and 1×10^6^ 0.5 ml−1 cells were incubated with live bacteria of S. carnosus strains expressing protein A or FnBPs at an MOI of 200. After 1 h of incubation cells were washed, stained with annexin V and propidium iodide (taking another hour) and then cell death was measured by flow cytometry. This figure shows one representative flow cytometric measurement. Here, we could detect a clear shift towards annexin V positive cells (positive for annexin V and negative for PI: sign for early apoptosis) resulting in 15–20% apoptotic cells. By contrast, stimulation with PVL did not cause apoptotic features (see Figure S2). These results indicate that strains, which express virulent surface proteins, can induce forms of programmed cell death. (1.79 MB TIF) [file ppat.1000715.s008.tif]
